# Bivalirudin or heparin: which anticoagulation strategy for critically ill cardiac surgery patients?

**DOI:** 10.1186/cc13287

**Published:** 2014-03-17

**Authors:** F Pappalardo, B Arnaez, M Celinska-Spodar, M Pieri, F Isella, S Silvetti, A Montisci, O Saleh, A Koster

**Affiliations:** 1 San Raffaele Scientific Institute, Milan, Italy; 2 Heart and Diabetes Centre, North Rhine-Westphalia Ruhr-University Bochum, Bad Oeyenhausen, Germany

## Introduction

Anticoagulation with unfractionated heparin in cardiac surgery patients has several limitations, and above all the risk of heparin-induced thrombocytopenia. Bivalirudin is a direct thrombin inhibitor, use of which in cardiac surgery patients has expanded in recent years. The aim of the study was to analyze two strategies for introducing bivalirudin in this setting (as secondary drug switching from heparin or as primary anticoagulant) and to evaluate clinical outcomes.

## Methods

Data from 100 propensity matched patients who received heparin (Group H, *n *= 50) or bivalirudin (Group B, *n *= 50), from January 2009 to January 2012, in a cardiac surgery ICU of a university hospital were analyzed. Bivalirudin was administered as either first-line or second-line drug after heparin discontinuation if heparin-induced thrombocytopenia was presumed.

## Results

Bivalirudin treatment was associated with a reduction of major bleeding (*P *= 0.05) compared with the control group. Interestingly, in an intention-to-treat analysis, patients receiving primary bivalirudin showed a significant reduction in minor bleeding (*P *= 0.04), and mortality (*P *= 0.01) compared with the secondary bivalirudin group, and similarly if compared with UFH and secondary bivalirudin patients (*P *= 0.01 and *P *= 0.05 respectively). Predictors of hospital mortality at multivariate analysis included urgent admission (OR = 2.7; 95% CI, 1.03 to 7.2; *P *= 0.04), septic shock (OR = 8.0; 95% CI, 2.26 to 28.7; *P *< 0.005) and primary therapy with UFH (OR = 19.2; 95% CI, 2.2 to 163.9; *P *= 0.007).

## Conclusion

Novel anticoagulant strategies might play a crucial role in critically ill cardiac surgery patients. In a propensity-matched population, our study showed that primary bivalirudin anticoagulation may reduce bleeding complications and mortality. Further studies are therefore warranted.

